# Changes in executive function and gait in people with mild cognitive impairment and Alzheimer disease

**DOI:** 10.1590/1980-57642021dn15-010006

**Published:** 2021

**Authors:** Natália Oiring de Castro Cezar, Juliana Hotta Ansai, Marcos Paulo Braz de Oliveira, Danielle Chagas Pereira da Silva, Francisco Assis Carvalho Vale, Anielle Cristhine de Medeiros Takahashi, Larissa Pires de Andrade

**Affiliations:** 1 Department of Physical Therapy, Universidade Federal de São Carlos – São Carlos, SP, Brazil.; 2 Graduate Program in Movement Sciences, Universidade Federal de Mato Grosso do Sul – Campo Grande, MS, Brazil.; 3 Graduate Program in Movement Sciences, Universidade Federal de Mato Grosso do Sul – Campo Grande, MS, Brazil.

**Keywords:** walking speed, longitudinal studies, cognition, cognitive dysfunction, aging, velocidade de caminhada, estudos longitudinais, cognição, disfunção cognitiva, envelhecimento

## Abstract

Changes in executive function and motor aspects can compromise the prognosis of older adults with mild cognitive impairment (MCI) and favor the evolution to dementia.

**Objectives:**

The aim of this study was to investigate the changes in executive function and gait and to determine the association between changes in these variables.

**Methods:**

A 32-month longitudinal study was conducted with 40 volunteers: 19 with preserved cognition (PrC), 15 with MCI and 6 with Alzheimer disease (AD). Executive function and gait speed were assessed using the Frontal Assessment Battery, the Clock-Drawing test and the 10-meter walk test. For data analysis, the Pearson product-moment correlation, two-way repeated-measures ANOVA, and chi-square were conducted.

**Results:**

After 32 months, an improvement in the executive function was found in all groups (p=0.003). At baseline, gait speed was slower in individuals with MCI and AD compared to those with PrC (p=0.044), that was maintained after the follow-up (p=0.001). There was significant increase in number of steps in all groups (p=0.001). No significant association was found between changes in gait speed and executive function.

**Conclusions:**

It should be taken into account that gait deteriorates prior to executive function to plan interventions and health strategies for this population.

## RESUMO.

Alterações na função executiva e nos aspectos motores podem comprometer o prognóstico de idosos com comprometimento cognitivo leve (CCL) e favorecer a evolução para demência.

**Objetivos:**

O objetivo deste estudo foi investigar alterações na função executiva e na marcha e determinar a associação entre alterações nessas variáveis.

**Método:**

Foi realizado um estudo longitudinal de 32 meses com 40 voluntários: 19 com cognição preservada (PrC), 15 com CCL e 6 com doença de Alzheimer (DA). A função executiva e a velocidade da marcha foram avaliadas por meio de bateria de avaliação frontal, do teste de desenho do relógio e do teste de caminhada de 10 metros. Para a análise de dados, o coeficiente de correlação produto-momento de Pearson, ANOVA de medidas repetidas bidirecional e o qui-quadrado foram realizados.

**Resultados:**

Após 32 meses, houve melhora na função executiva em todos os grupos (p=0,003). No início do estudo, a velocidade da marcha foi mais lenta nos indivíduos com CCL e DA em comparação com os PrC (p=0,044), que foi mantida após o acompanhamento (p=0,001). Houve aumento significativo no número de etapas em todos os grupos (p=0,001). Não foi encontrada associação significativa entre alterações na velocidade da marcha e função executiva.

**Conclusões:**

Deve-se levar em consideração que a marcha se deteriora antes da função executiva para planejar intervenções e estratégias de saúde para essa população.

## INTRODUCTION

Older adults with mild cognitive impairment (MCI) and Alzheimer disease (AD) experience changes in executive function (EF),^1^ ^–^ ^2^ which are more pronounced in the latter group.^3^ EF is a broad term related to planning, working memory, cognitive flexibility, monitoring, decision-making, and the ability to solve novel problems.^4^

A study that monitored older adults with preserved cognition (PrC), MCI, and mild to moderate AD for three years found that EF scores were significantly worse in those with AD compared to those with MCI, who, in turn, had worse scores than those with PrC.^5^ Considering the heterogeneous sample of the AD group (patients in the mild and moderate phases), studies assessing only older adults with mild AD are needed, since this population differs greatly from the population in the moderate phase of the disease with regard to cognitive and motor aspects.^6^ ^–^ ^8^

A relationship has been found between changes in gait and EF in older adults with cognitive impairment^9^ ^,^ ^10^ and those with AD in the mild and moderate phases.^6^ A poorer performance regarding EF measures is associated with a shorter step length and width as well as slower gait.^11^ In a study with a 23-month follow-up, reductions in cadence (number of steps per minute) and gait speed (GS) were associated with global cognitive decline and diminished EF in older adults with PrC.^12^

Slow GS is a strong predictor of dementia.^13^ Older adults with MCI who have lower limb impairment are more likely to develop AD than those with MCI and preserved lower limb function.^14^ Moreover, GS is a potential marker for the early identification of MCI.^15^ ^,^ ^16^

Few longitudinal studies have analyzed the relationship between gait and EF in older adults with and without cognitive impairment or have performed comparative analyses of older adults with PrC, MCI, and mild AD. As early diagnosis is important to the prognosis of older adults with MCI and its progression to dementia, the present study was conducted to identify changes in motor aspects and EF in this population and determine which ones declines first. The prompt identification of cognitive and gait changes enables the establishment of preventive actions. Therefore, the results of the present longitudinal analytical study can contribute to the planning of future interventions to mitigate such changes and their consequences.

Therefore, the aim of the present study was to investigate changes in EF and gait in older adults with PrC,MCI, and mild AD over a 32-month period and to analyze the correlation between the changes in these two variables. The hypothesis was that those with greater cognitive impairment would demonstrate a greater worsening in EF and GS after 32 months. It was also believed that the 10-meter walk test would be strongly correlated with EF tests.

## METHODS

The present longitudinal analytical study was conducted with data from the “Brazilian longitudinal study about motor alterations in older adults with cognitive disorders”. This study received approval from the local Human Research Ethics Committee (certificate number: 72774317.7.0000.5504). All volunteers signeda statement of informed consent.

### Sample

The subjects were recruited through leaflets, posters, and local radio and television channels. In addition, older people attending the Center for Medical Specialties, Universidade Aberta da Terceira Idade (São Carlos – SP), and School Health Unit (Universidade Federal de São Carlos) were contacted. This is a convenience sample.

Community-dwelling adults aged 65 years old or older who could be contacted by telephone or at their residential address were eligible for the study. Inclusion criteria included ability to walk at least 12.4 m with or without the aid of gait-assistance device, availability to participate in the evaluations, and admission to one of the three groups: PrC, MCI or mild AD. Exclusion criteria were: other neurological diseases that interfered in cognition or mobility and associated medications(such as motor alterations after stroke, Parkinson disease, multiple sclerosis, Huntington disease, epilepsy, traumatic brain injury, and advanced or moderate-stage of dementia), and severe uncorrected audiovisual impairment that would hinder test performance. Moreover, after the 32-month follow-up, participants with unsuccessful telephone or residential contact, those who died, became wheelchair-bound or bedridden, were unable to continue in the study due to illness (*i.e*.,influenza, deep vein thrombosis, acute lumbosacral pain, etc.), those who moved to a different city, and thosenot interested in continuing the evaluations were also excluded from the study. The massive loss of the initial sample may have caused a significant bias in the research. This type of loss is commonly observed in longitudinal studies with this population. We sought to reduce losses by offering transportation to participants, telephone contact with participants during the period between assessments, obtaining contact information from family or friends in case of a change of address or telephone number. Three attempts were made when trying to contact participants before they were considered a dropout.

The diagnosis and phase of AD was confirmed by a single neurologist trained in the field of behavioral neurology, based on the National Institute on Aging and Alzheimer’s Association criteria.^17^ Only individuals with a score of 1 on the Clinical Dementia Rating (CDR) scale were included in the mild AD group^.18^ Participants classified as PrC obtained a normal score on the Mini-Mental State Examination (MMSE)^19^ and did not meet the criteria for MCI or dementia. For the diagnosis of MCI: cognitive complaint manifested by the participant or a caregiver (person who cares for the older adult for at least 12 h per day, four times a week); objective cognitive decline with a score of 0.5 on the CDR;^18^ normal general cognitive function for level of schooling assessed by the MMSE;^19^ and preserved functioning evaluated by the Pfeffer Scale.^20^ ^,^ ^21^ After 32 months, the participants were reclassified.

### Evaluations

**Figure 1:**
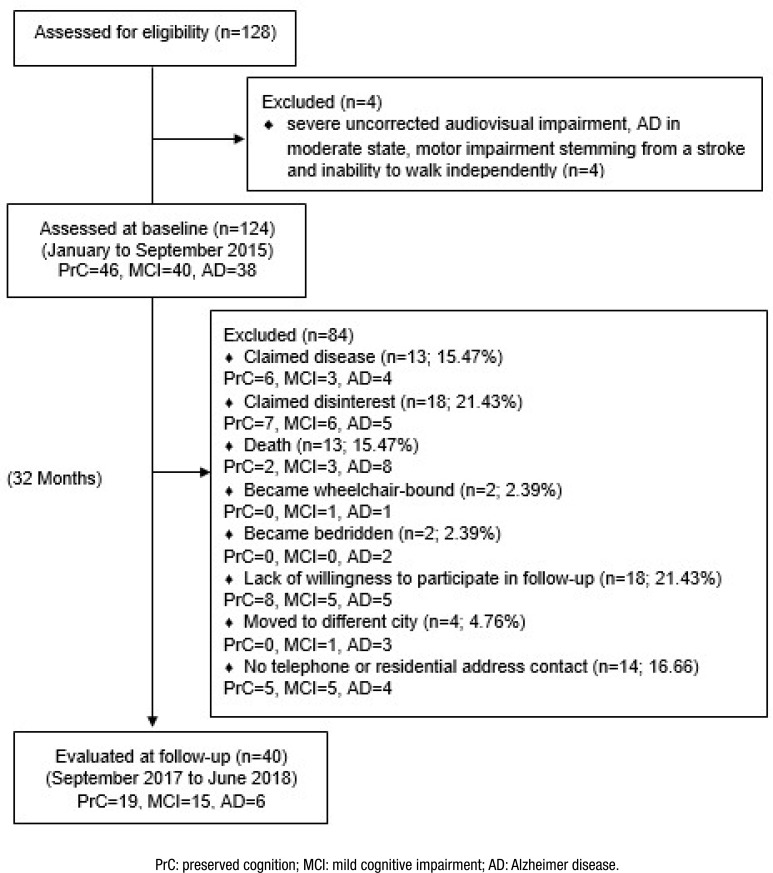
Sample flowchart.

Evaluations were performed on two occasions: baseline and follow-up (Figure 1). As there are studies research with shorter^22^ and intermediate^12^ ^,^ ^23^ follow-up, this study approached the longer period of 32 months. Participants were evaluated in the laboratory wearing comfortable clothes, closed-toe shoes, hearing aid and/or glasses, and no physical activity in the previous 24 hours. The tests were administered in a closed environment with a flat floor and minimal external visual and auditory stimuli. Evaluators were properly trained and explained all the tests to the participants in a simple, objective, and standardized way. When necessary, the participants had the help of a caregiver for the recording of the patient’s history (socio-demographic and health characteristics, such as age, gender, body mass index, schooling, use of medications in general and psychotropic drugs, and presence of disease in general, depression, and anxiety) and for the screening of depressive symptoms.^24^

The assessment of the EF was performed using the Frontal Assessment Battery (FAB) and clock-drawing test (CDT). The FAB is employed to evaluate frontal cognitive function, including EF. The maximum score is 18.^25^ ^,^ ^26^ Its inter-rater reliability is 0.87 and discriminant validity is 89.1%.^25^ The CDT is used to assess EF based on the design of an analog clock, for which the maximum score is 10. Its inter-rater reliability is 0.86.^27^ CDT has been translated, adapted, and validated for use in older adults in Brazil.^28^ In addition, CDT has good inter-examiner and test-retest reliability, high sensitivity and specificity, concurrent validity and predictive validity.^29^ The FAB and CDT were chosen because these tests detect changes in EF and are fast and easy to administer. Moreover, a strong association has been reported between frontal function and kinetic gait variables.^6^

GS was determined using the 10-meter walk test (10mWT) by video and stopwatch. On the 10mWT, participants are instructed to walk 12.4 m in a flat corridor at their usual pace. The initial and final 1.2 m are discarded to eliminate the components of acceleration and deceleration.^30^ The test was performed only once. The elements analyzed were the number of steps, GS, and cadence. Walk tests ranging from six to 15 m have good reliability and reproducibility and are valid for assessing physical mobility in a clinical or home setting.^30^ Inter-rater reliability for the walk test is 0.985.^31^ The 10mWT was chosen because it is widely used in the literature for the evaluation of GS. The minimal detectable change with 90% confidence for GS is 0.21 m/s.^32^

### Data analysis

Statistical tests were performed using the SPSS software, with a significance level of α=0.05. The Kolmogorov-Smirnov test was used to determine the normality of data distribution. One-way analysis of variance and the chi-square test were used to determine differences among the groups regarding the initial clinical and sociodemographic characteristics. When an overall group difference was significant, a post hoc independent Student’s t-test was performed.

Two-way repeated-measures ANOVA was used to determine the interaction between group and time with regard to EF and performance on the 10mWT. When a significant interaction was identified, analyses of the main simple effects were performed. Pearson’s correlation test was used to determine the correlation between the change in EF and GS between evaluations.

## RESULTS

One hundred and twenty-four volunteers were evaluated at baseline: 46 with PrC, 40 with MCI, and 38 with mild AD. After a 32-month follow-up, the dropout rate was 67.74% (n=84) due to deaths (15.47%), lack of willingness to participate in the follow-up evaluation (21.43%), change of address to a different city (4.76%), having become bedridden (2.39%), having become wheelchair-bound (2.39%), claimed disease (15.47%), disinterest (21.43%), and loss of contact via telephone or residence (16.66%). Thus, the final sample was composed of 19 older adults with PrC, 15 with MCI, and six with AD (Figure 1). There was a progression of two PrC participants to MCI and three MCI to DA, as well as a regression of six MCI to PrC after a 32-month follow-up.

Regarding sociodemographic characteristics at baseline, significant differences among the groups were found only for gender, total number of medications, and diseases. The MCI group had a higher number of women (93.3%) in comparison to the other groups. The MCI and mild AD groups took more medications and had more diseases compared to the PrC group (Table 1).

**Table 1:** Descriptive characteristics of the sample.

Characteristics (M±SD)	PrC group (n=19)	MCI group (n=15)	AD group (n=6)	p-value
Age (years)	72.7±6.7	72.8±5.4	77.6±4.1	0.195
Female gender, n (%)	10 (52.6)	14 (93.3)	3 (50.0)	**0.026** *
Body mass index (kg/m^2^)	28.5±5.7	29.8±3.9	26.1±4.0	0.296
Schooling (years)	7.9±4.2	5.2±3.9	6.5±5.0	0.207
Total number of medications	2.0±1.5	5.5±3.1#	5.5±2.9#	**<0.001** *
Use of psychotropics, n (%)	1 (5.3)	5 (33.3)	5 (83.3)	**<0.001** *
Total number of diseases	1.7±1.3	3.1±1.4#	3.8±1.3#	**0.003** *
Diagnosis of depression, n (%)	0(0)	0(0)	0(0)	-
Diagnosis of anxiety, n (%)	1(5.3)	1(6.7)	0(0)	0.816
GDS (0–15)	1.8±1.6	3.5±2.4	2.8±1.8	0.057

M±SD: mean±standard deviation; n (%): number of individuals (percentage); PrC: preserved cognition; MCI: mild cognitive impairment; AD: Alzheimer disease; kg/m^2^: kilogram/square meter; GDS: Geriatric Depression Scale; >5 points is suggestive of depression; ?10 points is almost always indicative of depression; >5 points should warrant follow-up comprehensive assessment;

* p<0.05 between groups;

# p<0.05 in comparison to PrC Group.

In the intragroup analysis of the change in GS on the 10mWT over time, a significant group versus time interaction was found (p=0.019). In the analysis of the main simple effects, both the PrC and mild AD groups had a worse performance after 32 months compared to baseline. A significant difference was found between the PrC and MCI groups at baseline (p=0.024), with a worse performance in the MCI group. Regarding the number of steps required to complete the 10mWT, no significant group versus time interaction was found, but a significant increase in the number of steps was found at follow-up in all groups (p=0.001) (Table 2).

**Table 2:** Performance on 10-meter walk test, Frontal Assessment Battery and clock-drawing test tests in older adults with preserved cognition, mild cognitive impairment and mild Alzheimer disease over 32 months (n=40).

Characteristics (M±SD)		PrC group (n=19)		MCI group (n=15)		AD group (n=6)		Time-group interaction p-value*	Time-group interaction Power*	Time p-value	Group p-value
		Baseline	Follow-up	Baseline	Follow-up	Baseline	Follow-up				
10mWT		Mean and SD		Mean an SD		Mean and SD					
	N. of steps	16.4±2.6	17.5±2.7	17.7±2.8	18.7±1.6	16.2±2.6	20.0±7.5	0.147	0.391	0.001*	0.354
	GS (m/s)	1.2±0.2	1.1±0.2	1.0±0.1	1.0±0.0	1.1±0.2 + #	0.9±0.2 #	**0.019** *	0.727	<0.001*	0.044*
	Cadence (steps/min)	113.0±13.6	113.2±11.8	103.9±15.5	108.8±7.9	106.0±14.7	102.4±13.1	0.285	0.264	0.828	0.123
FAB (maximum score=18)											
	Score	11.0±3.5	13.1±3.1	8.7±2.6#	10.1±3.1#	7.2±2.7#	9.5±5.2#	0.745	0.094	0.003*	0.006*
CDT (maximum score= 10)											
	Score	7.7±2.4	7.2±3.0	6.5±2.9	6.6±3.4	7.0±2.8	5.0±3.7	0.471	0.171	0.217	0.343

M±SD: mean±standard deviation; PrC: preserved cognition; MCI: mild cognitive impairment; AD: Alzheimer disease; GS: gait speed; 10mWT: 10-meter walk test; n°: number; FAB: Frontal Assessment Battery; CDT: clock-drawing test;

+ p<0.05 in comparison to PrC group at baseline;

# p<0.05 in comparison to PrC group;

* p<0.05; high score on FAB and CDT: high score on executive function.

No significant correlation was found between the change in EF (FAB) and change of GS in any of the groups. A correlation was found between the change in the FAB and the number of steps in the mild AD group and betweenthe change in the FAB and cadence in the PrC group (Table 3).

**Table 3:** Correlation between change in Frontal Assessment Battery and clock-drawing test tests and change in gait speed among older adults with preserved cognition, mild cognitive impairment, and mild Alzheimer disease

Correlation measurements	PrC group (n=19)	MCI group (n=15)	AD group (n=6)
DFAB with DGS	p=0.146	p=0.108	p=0.851
DFAB with DSTEPS	p=0.730	p=0.129	**p=0.001** r= -0.978
DFAB with DCADENCE	**p=0.042** r=0.472	p=0.627	p=0.664
DCDT with DGS	p=0.819	p=0.635	p=0.747
DCDT with DSTEPS	p=0.696	p=0.434	p=0.119
DCDT with DCADENCE	p=0.569	p=0.104	p=0.968

PrC: preserved cognition; MCI: mild cognitive impairment; AD: Alzheimer disease; FAB: Frontal Assessment Battery; CDT: clock-drawing test; GS: gait speed; Δ: final value–initial value; r: correlation coefficient.PrC: preserved cognition; MCI: mild cognitive impairment; AD: Alzheimer disease.

## DISCUSSION

In the present study, 32 months was not enough time for EF impairment in older adults with PrC, MCI, and mild AD. However, a decrease in GS at follow-up was found in those with PrC and mild AD. The findings suggest that the slowing of gait in individuals with PrC and mild AD is due to aging and cognitive impairment, respectively.

The deceleration in GS over time has been described in previous studies^33^ and GS has been associated with cognitive impairment.^13^ These findings are in agreement with Ojagbemi et al.,^23^ which reports a substantial change in GS associated with a reduction in cognitive performance after a 24-month follow-up.

In six-month follow-up studies of gait changes,^34^ ^–^ ^36^ no significant differences in GS were found in older adults with MCI. However, a 30-month follow-up study reports slower walking in older adults with amnestic MCI,^15^ which differs from the sample in the present investigation.

A slower GS was identified in older adults with MCI compared to those with PrC at baseline, but not at follow-up, possibly because GS in the PrC group has become slower over time, reflecting the influence of aging.^33^ ^,^ ^37^ ^–^ ^40^ It is believed that MCI participants have already reached a plateau in the GS decline. In addition, maybe changes on GS in the MCI group were not significant enough to be detected in a small sample size like this. Furthermore, possibly due to the heterogeneous evolution in the MCI group during follow-up, as some may have resumed normal cognition, remained stable or progressed to dementia. Although not confirmed by our data, studies suggest that the slowing of gait in individuals with PrC and mild DA is due to aging^33^ and cognitive impairment,^13^ respectively. The difficulty in assessing gait in older people is highlighted.

Although no significant difference was found among the groups, the AD group took the most number of steps on the 10mWT. As the power of this test is low, a larger number of individuals in the sample could have resulted in a significant p-value.

Regarding EF, no differences among groups or between times were found on the CDT and the change in FAB results over time was similar in all three groups. Moreover, significant differences in relation to FAB were found between the PrC and MCI groups as well as between the PrC and mild AD groups at baseline, whose differences were maintained at follow-up. The change in FAB was an improvement in the EF for all groups. Therefore, the CDT and FAB do not seem to be good markers to differentiate the evolution of cognition in these groups.

The FAB has discriminant validity as well as good internal consistency, interobserver reliability and convergent validity.^25^ However, there are no Minimum Detectable Change analyses to determine whether the change in score was clinically relevant. The standard deviations of the three groups ranged from 2.6 to 5.2 points and were reasonably high in the follow-up period compared to the values reported in other studies.^41^ ^,^ ^42^ Studies with larger samples may facilitate the conclusion of the findings.

In addition, the increase in the FAB was believed to have occurred for four reasons:

1. It was a group with mild AD, which mainly affects the temporal lobes.
2. It was AD rather than another form of dementia that affects the frontal lobe more.
3. The introduction of new pharmacological treatments (37.5%), physical activity (65%), and physical therapy interventions (57.5%) among the participants during the period between evaluations, given that some received their diagnosis during the study.
4. Due to possible learning of the instruments, since improvements were found in all groups (with no difference among groups).

Regarding the frontal functions, the analysis of the FAB results revealed no significant group versus time interaction. Improvements in FAB scores were found at follow-up in all groups (p=0.003). Moreover, significant differences were found between the PrC and MCI groups and between the PrC and mild AD groups at both evaluation times (p=0.006). No significant group versus time interaction was found with regard to cadence on the 10mWT or the CDT and no main significant group-time effect was found in these analyses (Table 2).

In studies by Ansai et al.,^43^ changes in EF were found at baseline and gait alterations were found at follow-up. However, changes in EF and GS do not go hand in hand, since motor decline precedes that of EF. GS is a good early marker of the development of MCI.^15^ ^,^ ^16^

No significant association was found between changes in the FAB and GS. However, associations were found between changes in the FAB and both the number of steps in the mild AD group and cadence in the PrC group. These findings are in agreement with the data reported by Pedroso et al.^44^ and Melo et al.,^45^ respectively. At follow-up, a decline in GS was found, while EF remained stable. Taylor et al.^22^ found an association between baseline GS and decline in EF in a 12-month period among older adults with dementia. In contrast, the present study included MCI and mild AD. Coelho et al.^6^ also found an association between GS and EF, but in a heterogeneous sample that included individuals with both mild and moderate AD. As these groups differ significantly in terms of cognitive and motor impairment,^6^ ^–^ ^8^ it is necessary to study them separately.

Two studies found an association between changes in gait and EF,^12^ ^,^ ^46^ however, the divergent results of the present investigation may have occurred because the authors used instruments to assess EF and gait variables different from those used in this study investigation. The literature shows that in addition to the consistency in the results and quality of the studies, there seems to be variations in the results according to the instrument chosen for the evaluation, sample size, population studied, and the evolution of cognitive impairment in the volunteers.^12^ ^,^ ^22^ ^,^ ^46^ ^,^ ^47^

As MCI and dementia become more prevalent with the increase in age, early diagnosis is essential. The results of the present study seem to indicate that slowing GS is a potential early marker of cognitive decline. Thus, rehabilitation professionals should perform periodic assessments of GS in older adults. Once decreased GS over time is detected, such individuals should be screened for cognitive decline to obtain an early diagnosis and timely intervention. Therefore, rehabilitation professionals should prioritize attention to gait variables during the clinical care of older adults with the aim of preventing their decline. If older adults with slower gait are admitted to a rehabilitation clinic, the main intervention of the care should be to promote an increase in GS.

A limitation of the present study was the use of a convenience sample. However, the diagnostic criteria were rigorous and based on the current literature.^17^ ^,^ ^21^ Moreover, the stringent, sophisticated methodology, extensive evaluation, and use of clinical instruments widely employed in the clinical practice strengthened the study. Another limitation was the small number of volunteers who participated in the follow-up evaluation. Longitudinal studies with this type of population pose a challenge, since the older adults with MCI and AD can exhibit physical and cognitive frailty, which makes data collection more difficult. In addition, caregivers are often over-burdened and may have little time and/or interest in participating in studies. However, the small sample size may have some impact on the lack of significance in some results.

Future researches should carry out population-based studies in developing countries, which have socioeconomic inequalities and different health conditions, in order to offer greater reliability in the characterization of cognitive and motor impairment in these populations. It is fundamental to perform selective sampling that differentiates older adults with preserved cognition, those with subjective memory complaints, those with MCI and its subtypes and those with AD and its different phases. It is also important to standardize the use of other common evaluation instruments of gait and/or EF to compare cognitive profiles, such as the Timed up and Go test. Finally, it is necessary to reproduce these analyses in larger samples so that loss to follow-up does not interfere with the results.

As conclusion, gait of older adults with PrC and mild AD slowed down in 32 months and, over the years, this group needs to take more steps to cover the same distance. The same period was insufficient to detect deficits in EF in the PrC, MCI, and AD groups, suggesting that gait changes occur in older adults before EF are affected. This study contributes to the field of research in older adults with cognitive impairment and offers a theoretical foundation for the planning of interventions and health promotion strategies for this population.
